# Crystal Structures of d-Lyxono-1,4-lactone and Its *O*-Tosyl Derivative

**DOI:** 10.3390/molecules30020287

**Published:** 2025-01-13

**Authors:** Anna Sosnowska, Jarosław Chojnacki, Justyna Samaszko-Fiertek, Janusz Madaj, Barbara Dmochowska

**Affiliations:** 1 Faculty of Chemistry, University of Gdansk, Wita Stwosza 63, 80-308 Gdansk, Poland; a.sosnowska.413@studms.ug.edu.pl (A.S.); j.samaszko-fiertek@ug.edu.pl (J.S.-F.); 2 Department of Inorganic Chemistry, GdańskTech, G. Narutowicza 11/12, 80-233 Gdansk, Poland; jaroslaw.chojnacki@pg.edu.pl

**Keywords:** D-lyxono-1,4-lactone, *O*-tosyl derivative, X-ray crystallography, Hirshfeld surface, NMR

## Abstract

γ- and δ-lactones were formed by bromine oxidation of commercially available D-lyxose, as confirmed by IR analysis. The former was isolated, and its structure was confirmed by NMR spectra and X-ray analysis. In this structure, the presence of both intermolecular and intramolecular hydrogen bonds was found. Intermolecular interactions in the crystal were illustrated using the Hirshfeld surfaces. Due to steric reasons, 3,5-*O*-isopropylidene-d-lyxono-1,4-lactone was formed, which in a further step led to the formation of a 2-*O*-tosyl derivative. Its structure was confirmed by X-ray diffraction analysis. The additional ring of the *O*-isopropylidene derivative caused the lactone ring to change conformation to *^3^E*. In the crystal structure of this compound, only C-H⸱⸱⸱O intermolecular interactions were present, as confirmed by the Hirshfeld surface analysis.

## 1. Introduction

Lactones are defined according to the IUPAC recommendations as “cyclic esters of hydroxy carboxylic acids, containing a 1-oxacycloalkan-2-one structure, or analogs having unsaturation or heteroatoms replacing one or more carbon atoms of the ring” [1]. Due to the position of the hydroxyl group participating in the formation of the lactone ring relative to the carboxyl group, we distinguish α, β, γ, and δ-lactones that differ in the size of the ring. Due to the stability of the ring, the most common lactones in nature have five- and six-membered rings, i.e., γ- and δ-lactones [2,3]. Of particular importance due to their biological activity are 1,4-lactones (γ-lactones). It is estimated that over 3000 of these types of lactones occur in nature [4]. Compounds containing a γ-lactone ring exhibit a number of very interesting biological properties, including antimicrobial [5,6,7,8,9,10,11,12], antimalarial [7,13,14,15], anti-inflammatory [16,17,18,19,20,21,22,23,24,25,26], and anticancer [27,28,29,30,31,32,33,34,35,36,37]. γ-Lactones are also found among the approved broad-spectrum drugs [38,39,40,41,42,43,44,45].

Sugars are a group of compounds whose unquestionable advantage is their relatively easy bioavailability and renewable nature as substrates. Sugar lactones are common compounds, and their laboratory synthesis is mainly based on the selective oxidation of the aldehyde group in aldoses, e.g., using bromine [46]. Sugar lactones with five-membered rings (γ-lactones) are generally more thermodynamically stable and predominate in products over lactones with six-membered rings (δ-lactones) [47]. The well-known structure of these compounds and the defined chirality of individual carbon atoms mean that they have long been widely used in organic synthesis. There are many examples of the use of sugar lactones for the synthesis of a number of compounds, such as surfactants [48,49,50,51,52,53,54], *C*-glycosyl compounds [55,56,57,58,59,60,61], iminoalditols [62,63,64,65], thiosugars [66,67,68], or compounds with specific bioactivity [69,70,71,72,73].

Despite the development of spectroscopic techniques, especially nuclear magnetic resonance (NMR), X-ray diffraction still remains the best source of information on the structure of chemical compounds, including, of course, sugar lactones. Over the years, a number of crystallographic structures of sugar lactones, including unsubstituted aldonolactones [74,75,76] and a number of derivatives of such lactones [77,78,79,80,81,82,83,84], have been recorded. Due to difficulties in obtaining suitable crystals of pentose γ-lactones with free hydroxyl groups, only one such structure for D-ribono-1,4-lactone has been described in the literature, measured under very specific conditions of −150 °C [85]. The crystallographic structures of other γ-lactones containing free hydroxyl groups such as 3-deoxy-2-*C*-hydroxymethyl-d-*erythro*-pentono-1,4-lactone [86] or 1,4-lactone obtained from tri-*O*-acetyl-d-glucal [87] have been described in the literature. It should be noted, however, that the former is not a derivative of a naturally occurring sugar, while the latter, due to the presence of a double bond in the lactone ring flattening its conformation, has a completely different structure.

In recent years, crystallographic analysis has been expanded to include the possibility of additional studies of intermolecular interactions based on Hirshfeld surface analysis. There are many interesting examples in the literature of the use of these analyses to study a number of different types of intermolecular interactions [88]. However, in the case of sugar compounds, we were able to find only one literature reference using this method [89], which prompted us to extend our research by analyzing the Hirshfeld surface of the crystallographic structures we obtained.

The originally planned goal of the work carried out was to obtain a suitable d-lyxono-1,4-lactone substrate containing a good leaving *O*-tosyl group at the terminal carbon atom, which could be used in the quaternization reaction for the synthesis of a series of quaternary ammonium salts. As a result of the performed syntheses, it turned out that instead of the expected 5-*O*-tosyl derivative, we obtained a 2-*O*-tosyl derivative.

## 2. Results and Discussion

As studies show, γ-lactones in which the ring is substituted with a methyl or methylene substituent, especially in the α position, are characterized by special biological activity [4]. Considering the relatively easy bioavailability of pentoses and the ease of their transformation into the corresponding lactones, it seems that they can be an interesting source of research on this type of compounds.

For many years we have been involved in the synthesis of quaternary ammonium salts derived from sugars [90,91]. As part of this work, we managed to obtain and perform crystallographic measurements of a number of compounds containing five-membered rings of furanose or oxolane character in their structure. Continuing this work, we attempted to obtain quaternary ammonium salts containing a γ-lactone ring. As a result of this work, we present the synthesis and structural studies of D-lyxono-1,4-lactone and its *O*-tosyl derivative.

**Scheme 1:**
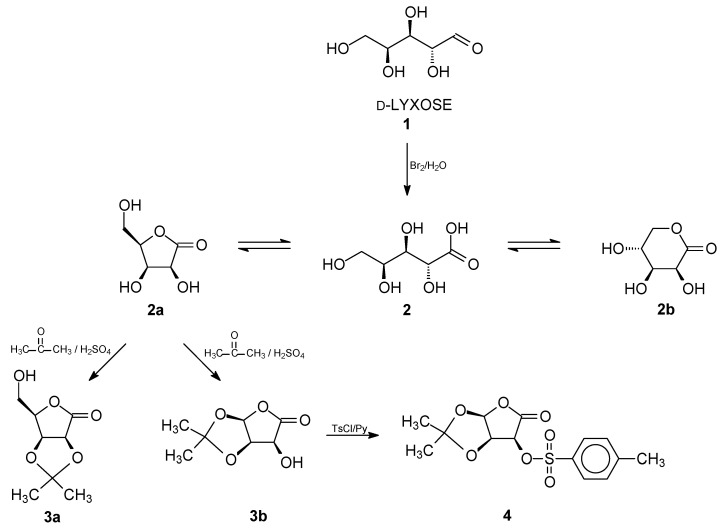
Synthesis of D-lyxono-1,4-lactone and its 2-*O*-tosyl derivative.

For the synthesis of D-lyxono-1,4-lactone and its derivative, we used D-lyxose as a substrate, which we oxidized by the classical method using bromine water [92]. Details of the syntheses performed are presented in Scheme 1. In aqueous solution, D-lyxose forms a state of thermodynamic equilibrium in which, in addition to cyclic forms, a chain form also occurs. The aldehyde group present in it can be oxidized to a carboxyl group by reaction with bromine water, resulting in the formation of D-lyxonic acid. Under appropriate conditions it can form a mixture of two lactones, containing a five-membered ring (γ-lactone, **2a**) and a six-membered ring (δ-lactone, **2b**), resulting from intramolecular esterification.

**Figure 1:**
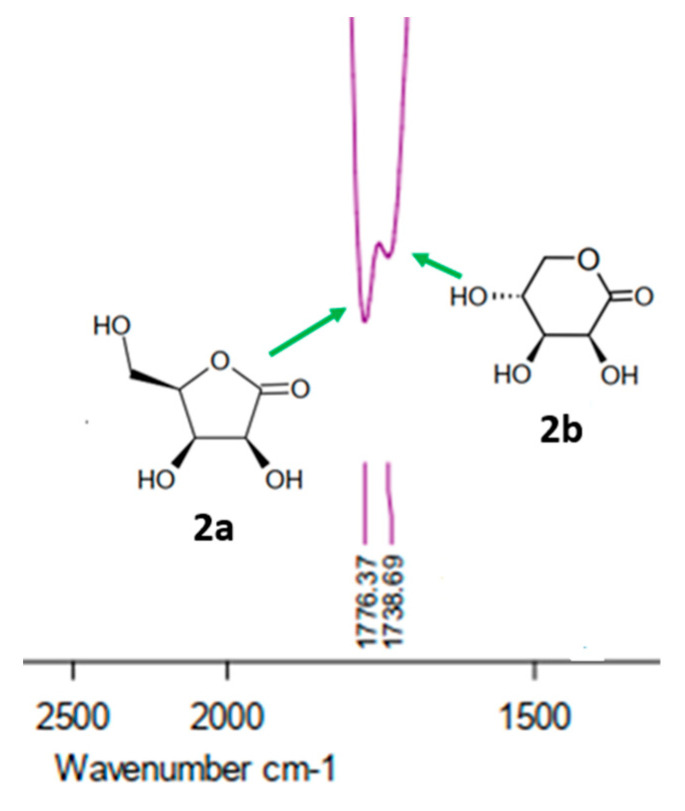
Fragment of the IR spectrum of the mixture of compounds **2a** and **2b** (the full spectrum is provided in the Supplementary Materials Figure S1).

Their presence is confirmed by the recorded IR spectrum (Figure 1).

**Figure 2:**
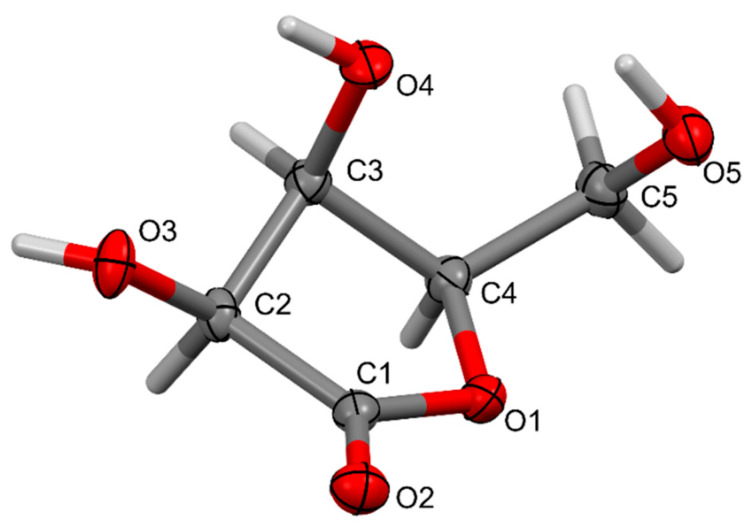
Molecular structure of compound **2a**, showing the atom-labeling scheme. Displacement ellipsoids are drawn at the 50% probability level and H atoms are shown in the capped sticks model.

According to the literature data [93], due to the larger ring size of δ-lactones, the vibration band of the C=O bond is located at the values of 1750–1735 cm^−1^, whereas for γ-lactones this band appears at the wave number values of 1780–1760 cm^−1^. In order to separate both isomers, crystallization from ethyl acetate was performed, resulting in the formation of white crystals of γ-lactone with a melting point of 105.5–106.0 °C (lit. [94] 110–111 °C). For this compound, 1D and 2D NMR spectra were recorded (see Supplementary Materials Figures S2–S5), which confirmed its structure. The structure of this compound was unequivocally confirmed by X-ray diffraction measurements. The structure determined in crystallographic studies is shown in Figure 2, and detailed data are included in Table 1.

**Figure 3:**
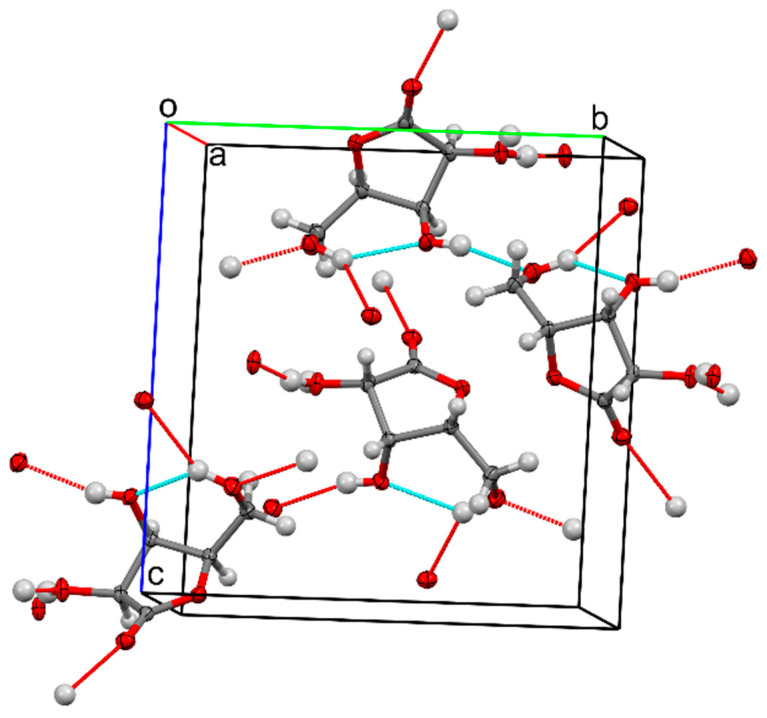
Crystal packing in structure **2a**. Only O-H⸱⸱⸱O type of hydrogen bonding shown as hashed blue lines (or red if the other part belongs to a symmetry equivalent molecule, not shown). Hydroxyl group O5-H5 forms intramolecular S(6) motif; other groups form intermolecular bonding.

The crystal packing of molecules is shown in Figure 3. Naturally, the structure optimizes hydrogen bonding of the O-H⸱⸱⸱O type and then also C-H⸱⸱⸱O (see Table 2 for details on hydrogen bonding parameters). All hydroxyl groups participate in the formation of hydrogen bonds. The terminal hydroxyl group forms an intramolecular hydrogen bond with the oxygen atom of the hydroxyl group at C3 (O5-H5···O4). In addition, this group and the hydroxyl group at C2 form intermolecular hydrogen bonds.

**Figure 4:**
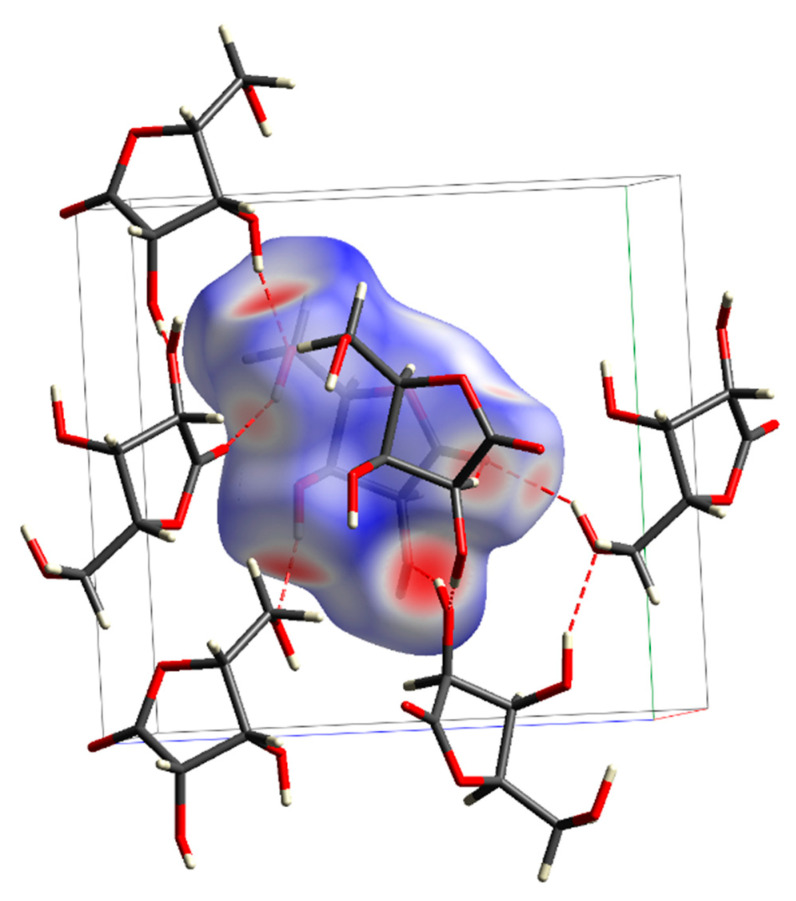
Main intermolecular interactions in structure **2a** illustrated by a Hirshfeld surface colored by d_norm, generated with CrystalExplorer. Red color indicates areas of strongest interactions.

As can be seen from the analysis of Figure 4, intermolecular interactions occur at sites of intermolecular hydrogen bonds, with the strongest being at the site of the shortest of them, O3-H3⸱⸱⸱O3.

**Figure 5:**
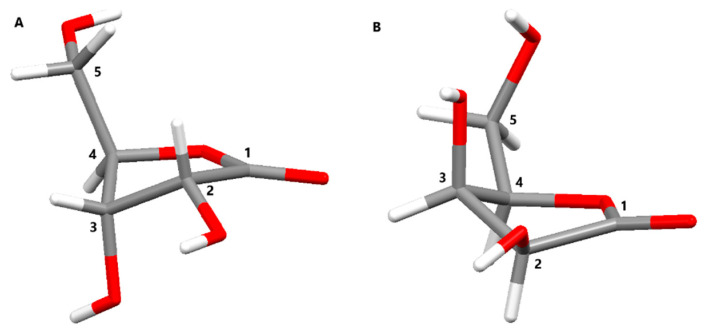
Conformations of the lactone ring in the crystal lattice of lactones with configuration (**A**) D-*ribo* ^2^*T*_3_ [85], (**B**) D-*lyxo* ^3^*T*_2_.

Due to the difficulties in crystallization of unsubstituted pentose γ-lactones, there is very little information about their crystallographic structure in the literature. Such a structure was measured at −150 °C for D-ribono-1,4-lactone [85]. This derivative crystallized in the *P*2_1_2_1_2_1_ space group of orthorhombic crystal system; the ring conformation was between *E*_3_ and ^2^*T*_3_ (Figure 5). The C=O bond length was 1.203(1) Å. In the case of compound **2a**, it crystallizes in the same space group; however, the ring conformation is ^3^*T*_2_ (twisted on C2-C3) and the C1-O2 carbonyl bond length reaches the value of 1.201(2) Å (all bond lengths and bond angles of compound **2a** are available in Supplementary Materials Tables S1–S3). Ring puckering parameters (Cramer & Pople) are presented in Table 3.

In both the D-*ribo* and D-*lyxo* conformations, the hydroxyl groups at C2 and C3 are in the *cis* configuration and are located on the same side of the ring. In both cases, the hydroxyl group at the C3 carbon atom tends to be arranged in the pseudo-axial position in the crystal lattice. Therefore, the difference in the configuration of the C2 and C3 carbon atoms in the D-*ribo* and D-*lyxo* lactones entails a change in the conformation of the lactone ring.

**Figure 6:**
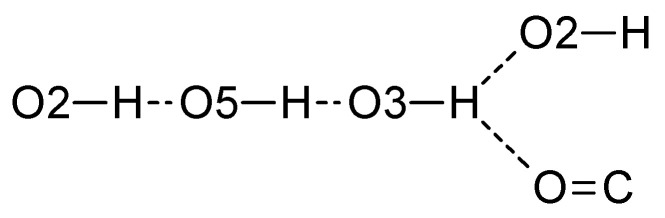
The hydrogen bonding scheme in crystal structure of D-ribono-1,4-lactone [85].

The hydrogen bond network in the D-*ribo* γ-lactone crystal (Figure 6) is different from that in the D-*lyxo* structure obtained by us and is presented in Figure 3. It contains only intermolecular hydrogen bonds, and their lengths vary in the range from 1.73 to 2.84 Å, In the crystal lattice of compound **2a** there are both intra- and intermolecular bonds, and their lengths reach values from 1.87 to 2.40 Å.

The obtained D-lixono-1,4-lactone (**2a**) was subjected to the reaction forming the *O*-isopropylidene derivative and, regardless of the method used, a mixture of two products (**3a**, **3b**) were obtained, which could not be separated at this stage. Therefore, the decision was made to carry out *O*-tosylation and attempt to separate its products. The reaction mixture formed after the *O*-tosylation reaction contained two main components. Unfortunately, attempts to separate it using chromatography were unsuccessful. After dissolving in methanol, the solution was placed at a temperature of −20 °C, and after about 2 weeks, small amounts of white crystals were filtered off. The mass and NMR spectra appeared to confirm that this was the expected *O*-tosyl derivative containing an *O*-isopropylidene protection. However, analysis of chemical shifts in the ^1^H and ^13^C NMR spectra of compound **2a** and the obtained tosylation product raised some doubts whether it was the expected 5-*O*-tosyl derivative. A large increase in the chemical shift values of the H2 proton (see Table 4, all NMR spectra of compound **4** are included in the Supplementary Materials Figures S6–S9) and the C2 carbon atom could indicate that the tosyl substituent is located at the C2 carbon atom.

**Figure 7:**
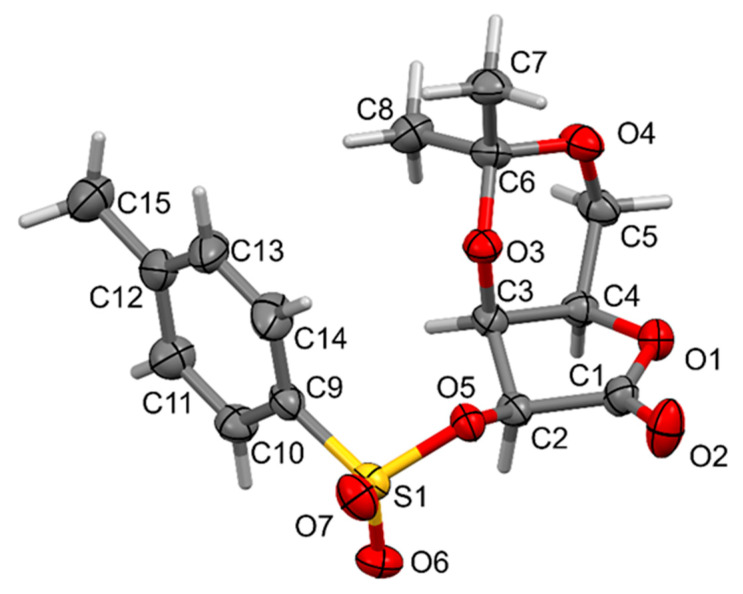
Molecular structure of compound **4**, showing the atom-labeling scheme. Displacement ellipsoids are drawn at the 50% probability level and H atoms are shown as rods.

**Figure 8:**
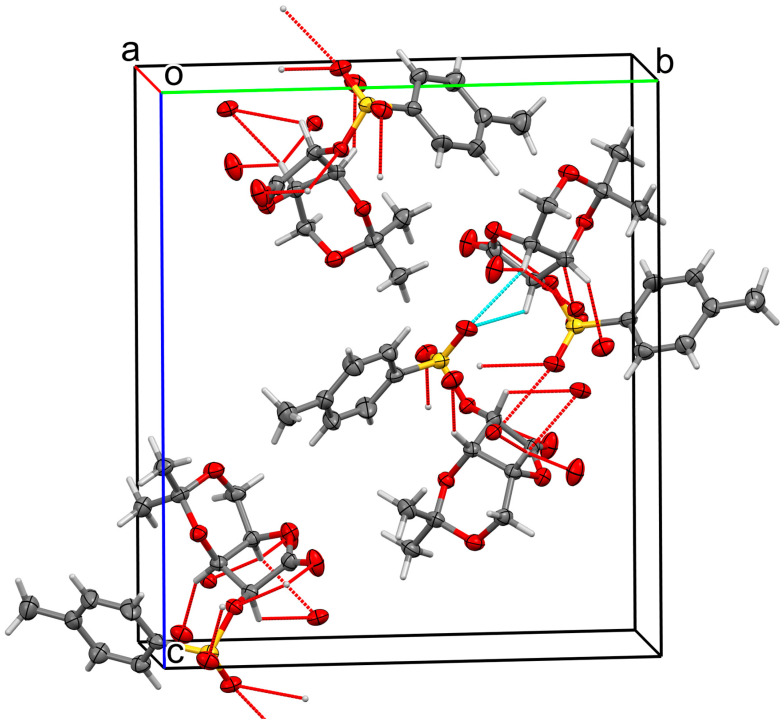
Crystal packing in structure **4**. Displacement ellipsoids are drawn at the 50% probability level; only weak intermolecular hydrogen bonding of C-H⸱⸱⸱O type is observed (see Table 5).

Ultimately, the unambiguous resolution of the issue of the structure of compound **4** was provided by X-ray structural analysis (Figure 7, detailed data are available in Table 1). The molecular packing in the crystal of compound **4** is shown in Figure 8.

**Figure 9:**
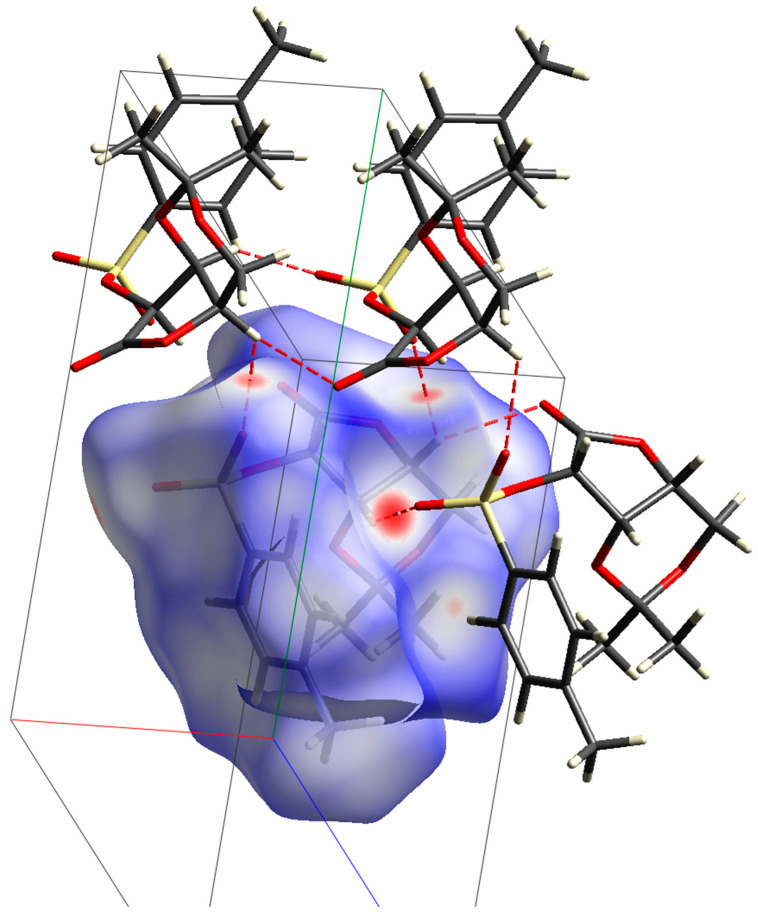
Main intermolecular interactions in structure **4** illustrated by a Hirshfeld surface colored by d_norm, generated with CrystalExplorer. Red color indicates areas of strongest interactions.

Analysis of the Hirshfeld surface shown in Figure 9 shows that the strongest intermolecular interactions result from the interaction between one of the oxygen atoms of the sulfone group and the hydrogen atom located at the C3 carbon atom.

As seen in Figure 7, compound **4** is 3,5-*O*-isopropylidene-2-*O*-tosyl-d-lyxono-1,4-lactone, which was found to be the major product of the *O*-tosylation reaction. Like in the case of the unsubstituted lactone, it crystallizes in the orthorhombic system, and the lactone ring adopts the ^3^*E* conformation (see Table 2). The change in conformation of the lactone ring is a result of the formation of the dioxane ring, and thus the stiffening of the structure. The C1-O2 bond length reaches 1.189(8) Å and is shorter than in the case of the unsubstituted lactone (all bond lengths and bond angles of compound **4** are available in Supplementary Materials Tables S4–S6).

The reason for the preferential formation of the 3,5-*O*-isopropylidene derivative in the case of the D-*lyxo* lactone, as opposed to the 2,3-*O*-isopropylidene derivative in the case of the D-*ribo* lactone, can be explained by analyzing Figure 5. The formation of the isopropylidene group requires the *cis* orientation of the hydroxyl groups involved in its formation. In the *ribo* configuration, only the groups at carbon atoms C2 and C3 satisfy this condition. In the *lyxo* configuration, this condition is met by the hydroxyl groups at carbon atoms C2 and C3 and the groups at carbon atoms C3 and C5, which makes it possible in the latter case to form two isomeric derivatives, 2,3- and 3,5-*O*-isopropylidene. However, for steric reasons, the arrangement of the hydroxymethyl group on the same side of the lactone ring as the hydroxyl groups at carbon atoms C2 and C3 favors the formation of the 3,5-*O*-isopropylidene derivative. This makes compound **4** the main product of the *O*-isopropylidene derivative formation and subsequent *O*-tosylation reaction.

## 3. Materials and Methods

### 3.1. General Section

D-Lyxose, *CAS* [*1114-34-7*], was purchased from Biosynth Ltd. (Compton, UK).

### 3.2. NMR Measurements

All measurements were carried out on a Bruker 500 MHz spectrometer. All spectra were recorded at a controlled temperature of 298 K using a TXI inverse probe. The obtained spectra were processed and analyzed with the usage of TopSpin 3.2 (Bruker BioSpin GmbH, Mannheim, Germany) software.

### 3.3. Mass Spectrometry

MALDI-TOF (Matrix-Assisted Laser Desorption/Ionization Time-of-Flight Mass Spectrometry) spectra were recorded using Bruker (Germany) Biflex III equipment. The matrixes used were DHB (2,5-dihydroxybenzoic acid) and CCA (α-cyano-4-hydroxycinnamic acid).

### 3.4. Infrared Spestroscopy

IR spectra were recorded using an IFS66 spectrometer from Bruker BioSpin GmbH, Mannheim, (Germany), performing Fourier transform infrared spectra with a resolution from 0.12 cm^−1^ for solid, liquid, and gaseous samples in the entire range, i.e., MIR (4000–400 cm^−1^), FIR (700–4.0 cm^−1^). Spectra S53–S55 were recorded courtesy of Pro-Environment Polska Sp. z. o. o., which provided an FT-IR Spectrometer, model: Spectrum Two with ATR attachment (Spectrum Two FT-IR Spectrometer with LiTaO_3_ Detector, PerkinElmer, Waltham, MA, USA).

### 3.5. Single-Crystal X-Ray Diffraction

Diffraction intensity data for **2a** and **4** were collected on an IPDS 2T dual-beam diffractometer (STOE & Cie GmbH, Darmstadt, Germany) at 120.0(2) K with MoKa radiation of a microfocus X-ray source (GeniX 3D Mo High Flux, Xenocs, Sassenage, 50 kV, 1.0 mA, and λ = 0.71069 Å). Investigated crystals were thermostated under a nitrogen stream at 120 K using the CryoStream-800 device (Oxford CryoSystem, Hanborough Business Park, Hanborough House 25, Long Hanborough OX29 8LH, UK) during the entire experiment.

Data collection and data reduction were controlled by using the X-Area 1.75 program (STOE, 2015). Due to low absorption coefficient, no absorption correction was performed for structure **2a**. The structures were solved using intrinsic phasing implemented in SHELXT and refined anisotropically using the program packages Olex2 [95] and SHELX-2015 [96,97]. Positions of the C–H hydrogen atoms were calculated geometrically taking into account isotropic temperature factors. All H atoms were refined as riding on their parent atoms with the usual restraints.

Absorption correction was performed for structure **4** by the scaling of reflection intensities. Afterwards, a spherical absorption correction was performed within STOE LANA program.

Crystallographic data for all structures reported in this paper have been deposited with the Cambridge Crystallographic Data Centre as supplementary publication No. CCDC 2387009-2387010. The data can be obtained free of charge from The Cambridge Crystallographic Data Centre via www.ccdc.cam.ac.uk/ structures (access on January 9, 2025). Geometry parameters were calculated in PLATON program [98] (see http://www.platonsoft.nl/, access on 9 January 2025), Hirshfeld surfaces have been generated using CrystalExplorer, ver. 21.5 [99].

### 3.6. d-Lyxono-1,4-Lactone (**2a**)

In a round-bottomed flask, D-lyxose (5.00 g, 33.35 mmol) and 5.50 g of potassium carbonate were placed. This mixture was dissolved in water (60 mL) and stirred at 0 °C. Bromine (2.0 mL, 77.7 mmol) was added dropwise to the mixture. After 1 h, cooling was stopped and the mixture was acidified with 88% HCOOH to pH 3–4. After removal of the volatile components under reduced pressure to remove inorganic salts, the oil was dissolved in ethanol (30 mL) at room temperature. After filtration, the salts were washed with additional ethanol (20 mL). The combined ethanol layers were concentrated under reduced pressure. In total, 3.97 g (80.5% yield) of a mixture of products **2a** and **2b** was obtained, which was stored over a drying agent (CaCl_2_) at −20 °C. Then, 0.04 g was taken from this mixture and dissolved in boiling ethyl acetate (5 mL). After reaching room temperature, white crystals began to precipitate from the solution. Finally, crystallization was carried out at −20 °C and D-lyxono-1,4-lactone (**2a**) (0.019 g, 0.13 mmol, 39.3% yield) was obtained as white crystals [mp 105.5–106.0 °C (lit. [90] 110–111 °C)] while the more soluble compound **2b** remained in the solution. ^1^H NMR (500 MHz, D_2_O): 4.67 (*d*, 1H, *J_2,3_* 5.1, H-2), 4.50 (*dd*, 1H, *J_3,4_* 4.4, H-3), 4.56 (*dt*, 1H, *J_4,5_* 2.6, *J_4,5′_* 7.6, H-4), 3.89–3.81 (*m*, 2H, H-5 and H-5′); ^13^C NMR (125 MHz, D_2_O): 178.3 C-1, 81.6 C-4, 70.5 C-2, 69.5 C-3, 59.7 C-5.

### 3.7. 2,3-O-Isopropylidene-d-Lyxono-1,4-Lactone (**3a**)

#### 3.7.1. Procedure A

In a flask, D-lyxono-1,4-lactone (**2a**) (3.812 g 25.76 mmol) was dissolved in acetone (16.7 mL) and 2,2-dimethoxypropane (3.4 mL) and concentrated sulfuric acid (0.024 mL) were added. The contents of the flask were stirred for 50 min at room temperature. Then the mixture was neutralized with NaHCO_3_. After filtering off the precipitate, the filtrate was concentrated under reduced pressure (bath temperature ca. 37 °C) to obtain an orange–brown, thick oil. The resulting mixture of products **3a** and **3b** was dissolved in ethyl acetate at 40 °C and filtered to remove inorganic salts. The filtrate was concentrated to half the volume and left overnight at a temperature of about 3 °C. Them, 1.87 g of a mixture of products **3a** and **3b** was obtained in the form of a crystallizing light yellow oil.

#### 3.7.2. Procedure B

In a flask, D-lyxono-1,4-lactone (2a) (3.21 g, 21.68 mmol) was dissolved in acetone (48 mL), and 2,2-dimethoxypropane (7.8 mL) and 70% methanesulfonic acid (0.8 mL) were added. The resulting mixture was stirred for 20 h at room temperature. Then the mixture was neutralized with saturated NaHCO_3_ to pH about 6–7. After filtering off the resulting precipitate, the filtrate was concentrated under reduced pressure. The crude mixture of products **3a** and **3b** was dissolved in water (24 mL), extracted with diethyl ether (2 × 16 mL), and then the organic layer was dried with MgSO_4_. After filtering off the drying agent and concentrating, 0.84 g of the mixture of products **3a** and **3b** was obtained as a light yellow oil.

### 3.8. 2,3-O-Isopropylidene-5-O-tosyl-D-Lyxono-1,4-Lactone (**4**)

In the flask: 1.87 g of the previously obtained mixture of products **3a** and **3b** was dissolved in cold pyridine (18.6 mL). Then, tosyl chloride (3.94 g, 20.66 mmol) was added in small portions over about 1.5 h, while maintaining the temperature in the range of 0–2 °C. After the addition of tosyl chloride was completed, the mixture was stirred for another hour at 0 °C and then left for 20 h at 5 °C. After adding 30 mL of CH_2_Cl_2_ to the mixture, the solution was extracted with water (10 mL), 10% aqueous HCl solution (6 mL), and saturated aqueous NaHCO_3_ solution (6 mL). The organic layer was dried with MgSO_4_ and decolorized with activated carbon. After filtration, the mixture was concentrated under reduced pressure to obtain 1.49 g of the mixture of products as a yellow oil. The resulting oil was dissolved under reflux in methanol (15 mL). After cooling, the solution was concentrated until crystals precipitated. The mixture was left at about 3 °C. Them, 3,5-*O*-isopropylidene-2-*O*-tosyl-D-lyxono-1,4-lactone (0.08 g, 0.23 mmol) (**4**) was obtained as white crystals (mp 207.5–208.5 °C). ^1^H NMR (500 MHz, CDCl_3_): 5.4 (*d*, 1H, *J_2,3_* 4.5, H-2), 4.66 (*dd*, 1H, *J_3,4_* 2.5, H-3), 4.19 (*dd*, 1H, *J_4,5_* 2.5, *J_4,5′_* 4.8, H-4), 4.13–4.06 (*m*, 2H, H-5 and H-5′); ^13^C NMR (125 MHz, CDCl_3_): 168.7 C-1, 74.9 C-2, 70.0 C-4, 66.8 C-3, 57.8 C-5, 28.0 *CH_3_*-Ph, 21.9, and 19.0 *CH_3_*-C.

## 4. Conclusions

As a result of synthetic work on obtaining the *O*-tosyl derivative of D-lyxono-1,4-lactone, it was possible to isolate and obtain compounds **2a** and **4** in the form of crystals, for which crystal structures were determined. The tendency for the hydroxyl group at the C3 carbon atom to be in a pseudo-axial position caused the lactone ring to be in the *^3^T_2_* conformation in the crystal lattice of compound **2a**. For steric reasons, in the *O*-isopropylidene reaction, the formation of a six-membered (dioxane) ring with hydroxyl groups at carbon atoms C3 and C5 was preferred over the formation of a five-membered ring with groups at carbon atoms C2 and C3. This ultimately led to the formation of derivative **4**, in which the *O*-tosyl group was present at the C2 carbon atom, which was confirmed by X-ray diffraction studies. The presence of the additional ring caused a change in the conformation of the lactone ring to *^3^E*. In both crystal structures, intermolecular interactions were observed that are well illustrated by Hirshfeld surfaces.

## Figures and Tables

**Table 1:** Crystal data and structure refinement parameters for structures **2a** and **4**.

	(2a)	(4)
Crystal data		
CCDC	2387009	2387010
Chemical formula	C_5_H_8_O_5_	C_15_H_18_O_7_S
*M*_r_	148.11	342.35
Crystal system, space group	Orthorhombic, *P*2_1_2_1_2_1_	Orthorhombic, *P*2_1_2_1_2_1_
Temperature (K)	120	120
*a*, *b*, *c* (Å)	4.8905 (2), 10.7479 (6), 11.3738 (6)	5.9288 (2), 15.1369 (5), 17.5364 (8)
*V* (Å^3^)	597.84 (5)	1573.78 (10)
*Z*	4	4
Radiation type	Mo *K*a	Mo *K*a
m (mm^−1^)	0.15	0.24
Crystal size (mm)	0.43 × 0.05 × 0.04	0.45 × 0.04 × 0.03
Data collection		
Diffractometer	STOE *IPDS* 2T	STOE *IPDS* 2T
Absorption correction	none	Multi-scan STOE LANA
*T*_min_, *T*_max_	–	0.159, 0.993
No. of measured, independent and observed [*I* > 2s(*I*)] reflections	7596, 1617, 1520	7534, 2969, 2584
*R*_int_	0.032	0.080
(sin q/l)_max_ (Å^−1^)	0.686	0.608
Refinement		
*R*[*F*^2^ > 2s(*F*^2^)], *wR*(*F*^2^), *S*	0.034, 0.094, 1.04	0.073, 0.201, 1.08
No. of reflections	1617	2969
No. of parameters	101	211
H-atom treatment	H atoms treated by a mixture of independent and constrained refinement	H-atom parameters constrained
Dρ_max_, Dρ_min_ (e Å^−3^)	0.36, −0.17	0.37, −0.49
Absolute structure	Flack x determined using 588 quotients [(I+) − (I−)]/[(I+) + (I−)] (Parsons, Flack and Wagner, Acta Cryst. B69 (2013) 249–259).	Classical Flack method preferred over Parsons because s.u. lower.
Absolute structure parameter	0.5 (10)	0.3 (2)

**Table 2:** Hydrogen bonding parameters for **2a**.

Structure 2a				
*D*—H···*A*	*D*—H	H···*A*	*D*···*A*	*D*—H···*A*
O3—H3···O3 ^i^	0.83 (3)	1.87 (3)	2.6756 (10)	162 (3)
O4—H4···O5 ^ii^	0.77 (3)	2.07 (3)	2.8155 (18)	165 (3)
O5—H5···O2 ^iii^	0.91 (3)	2.39 (3)	3.1358 (18)	139 (2)
O5—H5···O4	0.91 (3)	2.18 (3)	2.8202 (18)	127 (2)
C2—H2···O2 ^iv^	1.00	2.29	3.269 (2)	164
C4—H4A···O1 ^v^	1.00	2.61	3.297 (2)	126
C5—H5A···O2 ^v^	0.99	2.55	3.535 (2)	172

Symmetry codes: ^i^ x − ½, −y+ ½, −z +1; ^ii^ −x + 2, y − ½, −z + 3/2; ^iii^ −x + 5/2, −y + 1, z+ ½; ^iv^ x − 1, y, z; ^v^ x − ½, −y + 3/2, −z + 1.

**Table 3:** Ring puckering parameters for **2a** and **4** (calculated by PLATON program).

Compound	Q(2)/Å	f(2)/°	d/°	Closest Conformation
**2a**	0.3953(17)	278.4(2)	15.5(2)	twisted on C2-C3
**4**	0.396(6)	284.5(8)	29.8(10)	envelope on C3

**Table 4:** Chemical shifts [ppm] of protons and carbon atoms in compounds **2a** and **4** (atom numbering as shown in Figure 2).

Compound	H2	H3	H4	H5 H5′	C1	C2	C3	C4	C5
**2a**	4.67	4.50	4.56	3.85	178.3	70.5	69.5	81.6	59.7
**4**	5.4	4.66	4.19	4.10	168.7	74.9	66.8	70.0	57.8

**Table 5:** Hydrogen bonding parameters for **4**.

Structure 4				
D—H···A	D—H	H···A	D···A	D—H···A
C3—H3···O7 i	1.00	2.40	3.290 (8)	149
C4—H4···O2 i	1.00	2.50	3.236 (8)	130
C4—H4···O6 ii	1.00	2.49	3.207 (7)	128

Symmetry codes: i: *x*−1, *y*, *z*; ii: *x*−1/2, −*y* + 3/2, −*z* + 1.

## Data Availability

Data are contained within the article and Supplementary Materials.

## Supplementary Materials

The following supporting information can be downloaded at: https://www.mdpi.com/article/10.3390/molecules30020287/s1.
