# Supplementary material for: Crystal Structures of d-Lyxono-1,4-lactone and Its O-Tosyl Derivative
Source: Molecules. 2025 Jan 13;30(2):287. doi: 10.3390/molecules30020287 (PMC11767691; doi:10.3390/molecules30020287)
Supplement: Supplementary file 1 [file molecules-30-00287-s001.zip › molecules-3409282-supplementary.pdf]

SUPPLENETARY MATERIALS

Fig. S1. IR spectrum of D-lyxono-1,4-lactone (**2a**) and D-lyxono-1,5-laktone (**2b**). 2

Fig. S2. ^{1}H NMR spectrum of D-lyxono-1,4-lactone (**2a**). 3

Fig. S3. ^{13}C NMR spectrum of D-lyxono-1,4-lactone (**2a**). 4

Fig. S4. COSY spectrum of D-lyxono-1,4-lactone (**2a**). 5

Fig. S5. HSQC spectrum of D-lyxono-1,4-lactone (**2a**). 6

Fig. S6. ^{1}H NMR spectrum of 3,5-*O*-isopropylidene-2-*O*-tosyl- D-lyxono-1,4-lactone (**4**). 7

Fig. S7. ^{13}C NMR spectrum of 3,5-*O*-isopropylidene-2-*O*-tosyl- D-lyxono-1,4-lactone (**4**). 8

Fig. S8. COSY spectrum of 3,5-*O*-isopropylidene-2-*O*-tosyl- D-lyxono-1,4-lactone (**4**). 9

Fig. S9. HSQC spectrum of 3,5-*O*-isopropylidene-2-*O*-tosyl- D-lyxono-1,4-lactone (**4**). 10

Table S1. Fractional atomic coordinates and isotropic or equivalent isotropic displacement parameters (Å^2) for compound **2a**. 11

Table S2. Atomic displacement parameters (Å^2) for compound **2a**. 12

Table S3. Geometric parameters (Å, º) for compound **2a**. 12

Table S4. Fractional atomic coordinates and isotropic or equivalent isotropic displacement parameters (Å^2) for compound **4**. 14

Table S5. Atomic displacement parameters (Å^2) for compound **4**. 15

Table S6. Geometric parameters (Å, º) for compound **4**. 16

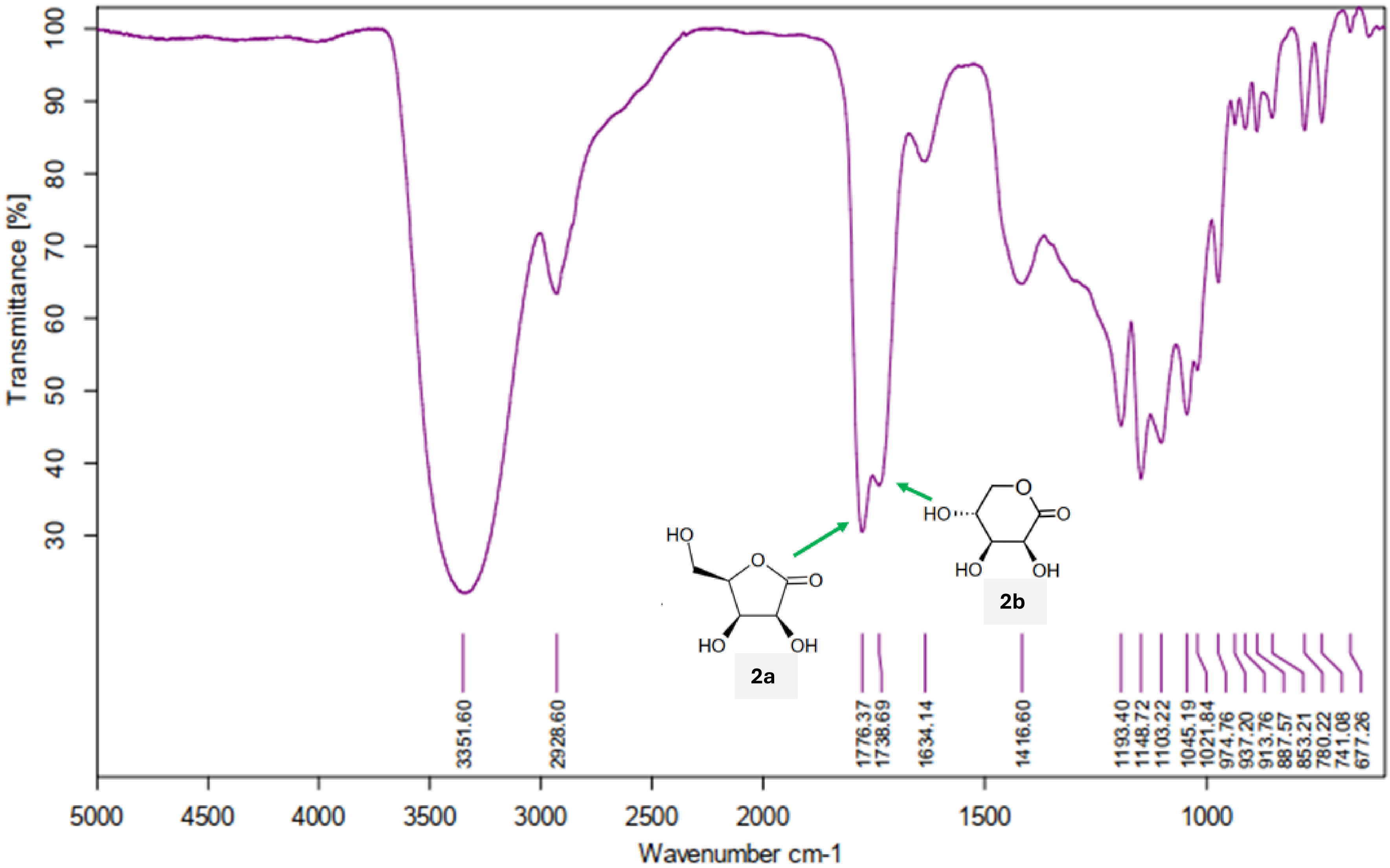

Fig. S1. IR spectrum of D-lyxono-1,4-lactone (**2a**) and D-lyxono-1,5-laktone (**2b**).

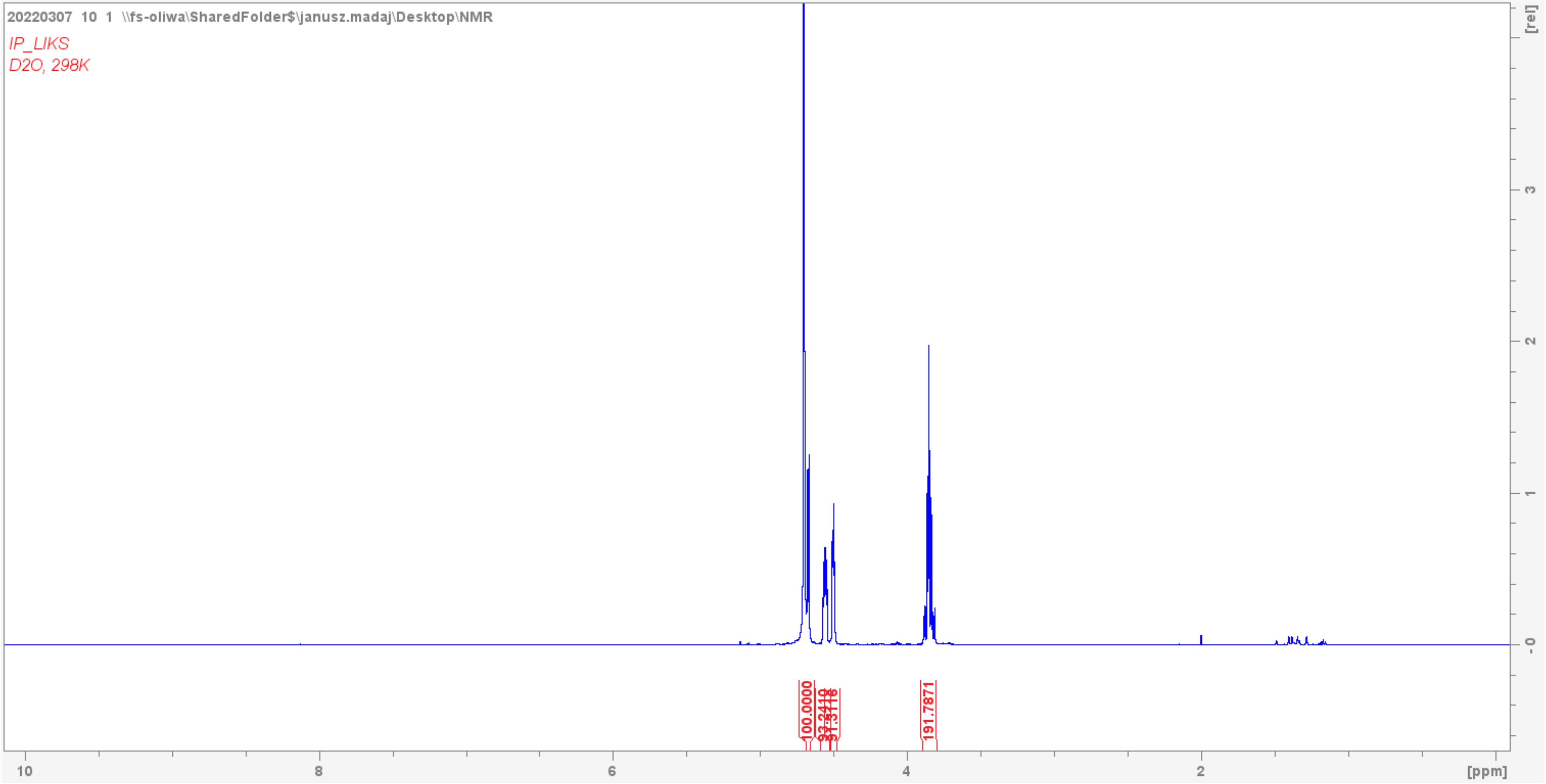

Fig. S2. ^{1}H NMR spectrum of D-lyxono-1,4-lactone (**2a**).

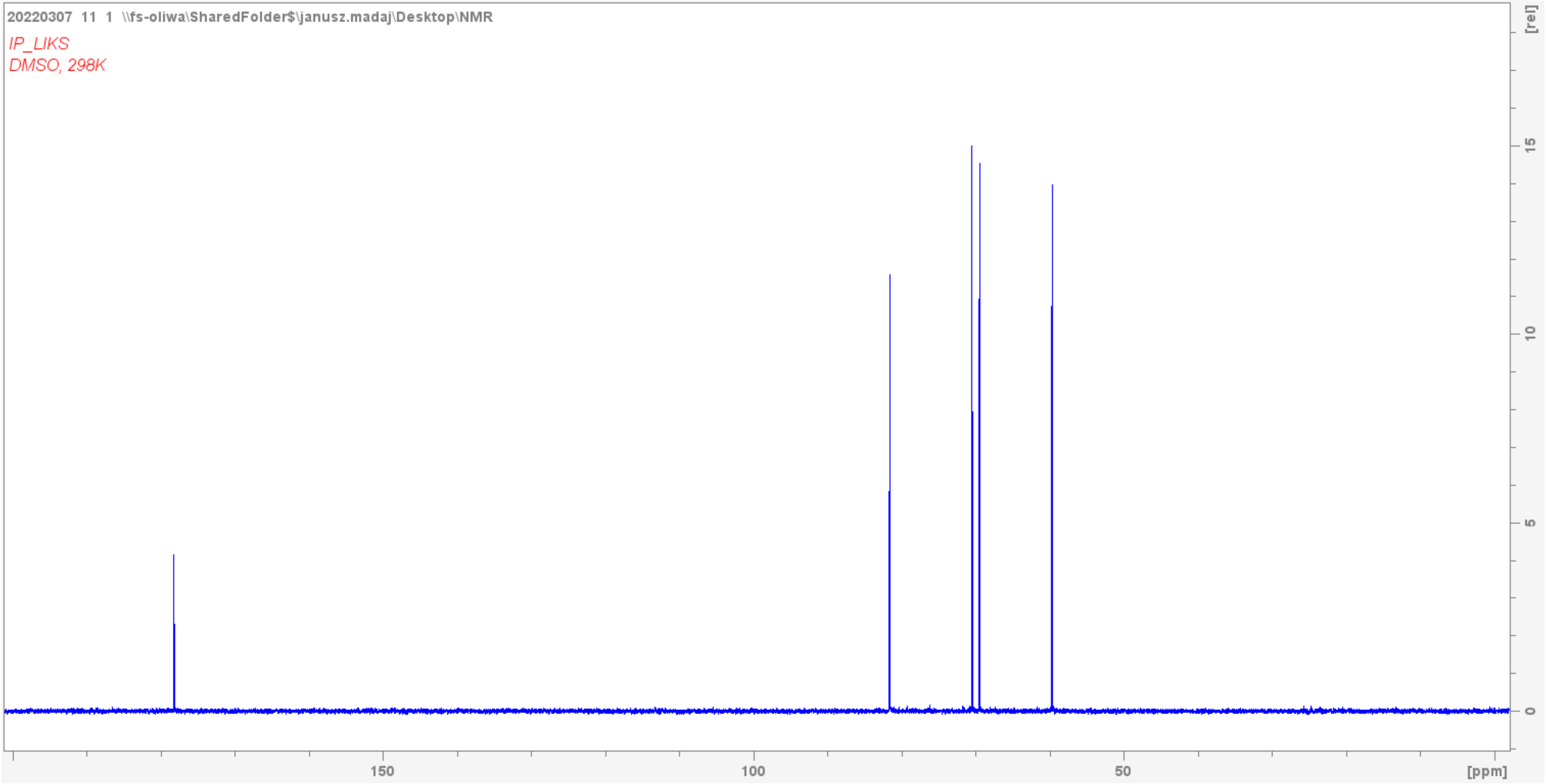

Fig. S3. ^{13}C NMR spectrum of D-lyxono-1,4-lactone (**2a**).

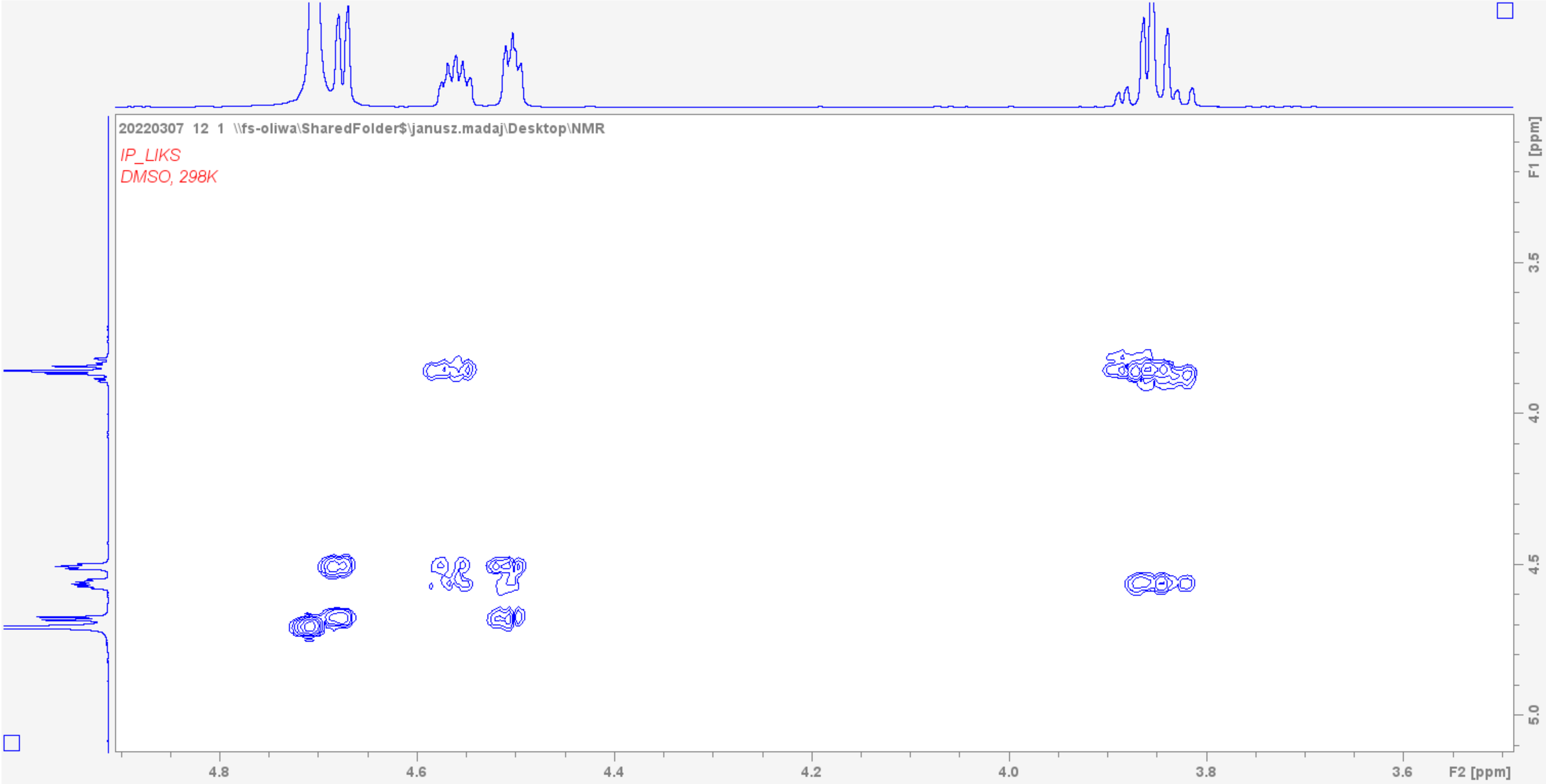

Fig. S4. COSY spectrum of D-lyxono-1,4-lactone (**2a**).

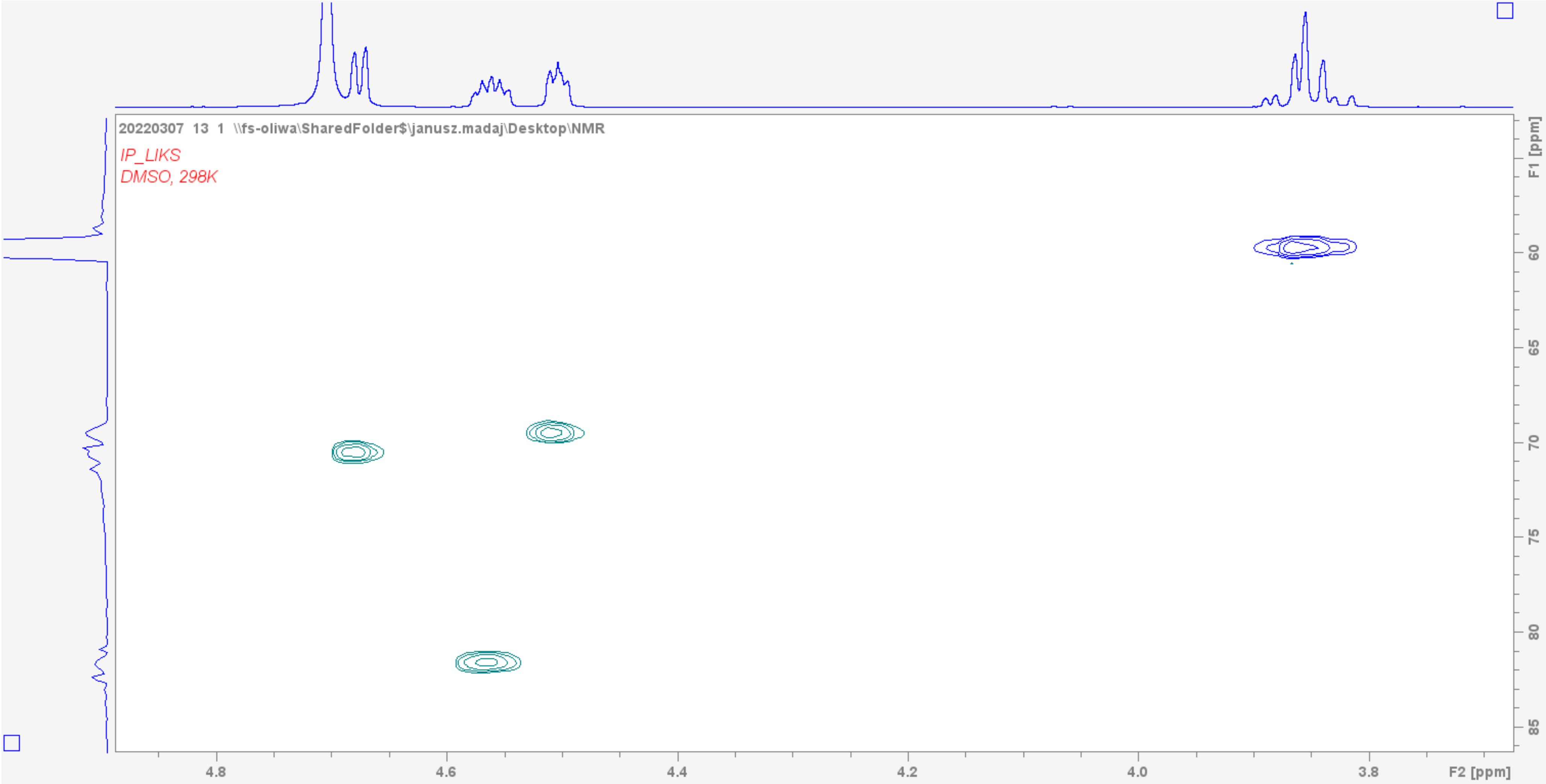

Fig. S5. HSQC spectrum of D-lyxono-1,4-lactone (**2a**).

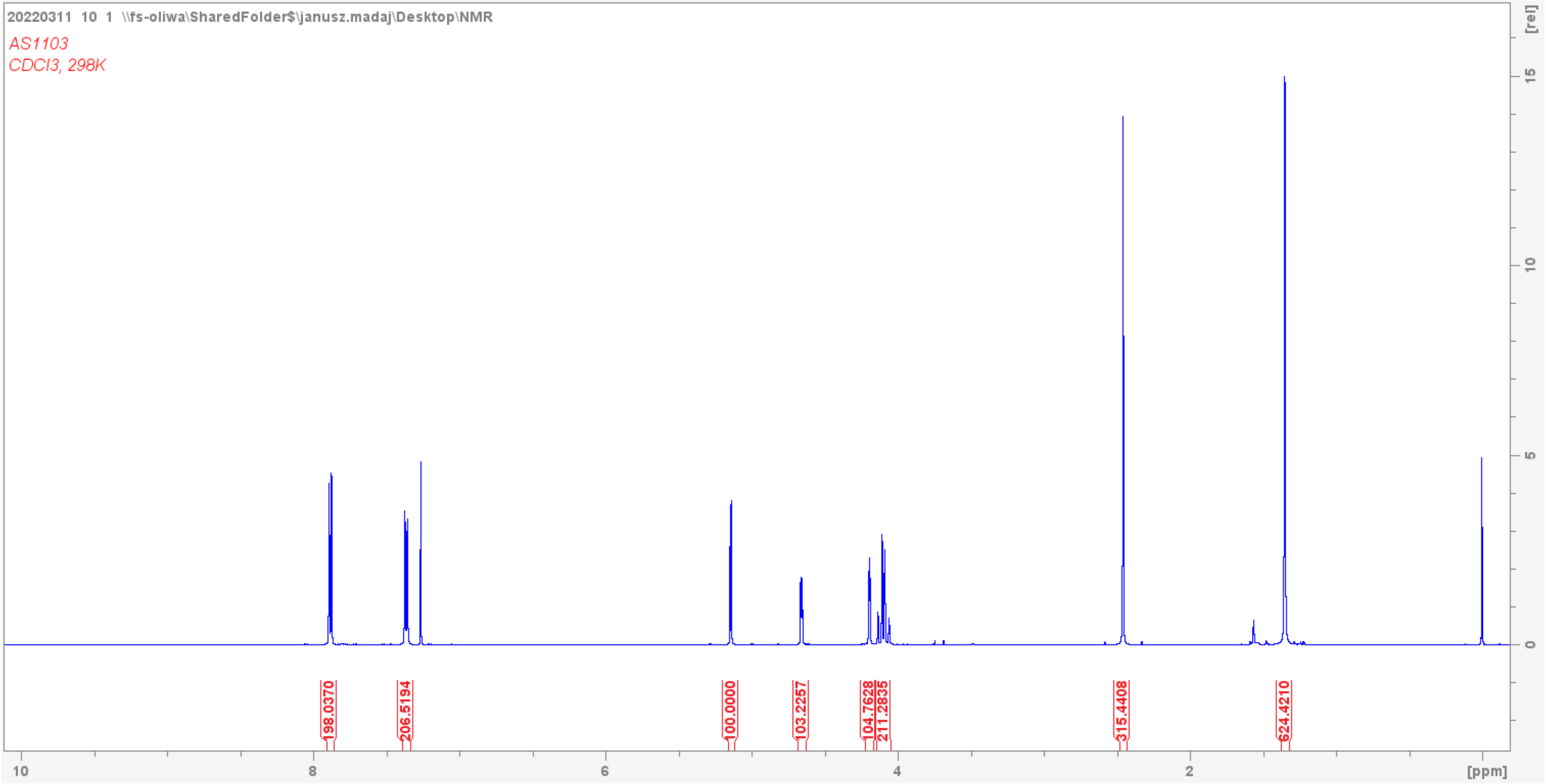

Fig. S6. ^{1}H NMR spectrum of 3,5-*O*-isopropylidene-2-*O*-tosyl- D-lyxono-1,4-lactone (**4**).

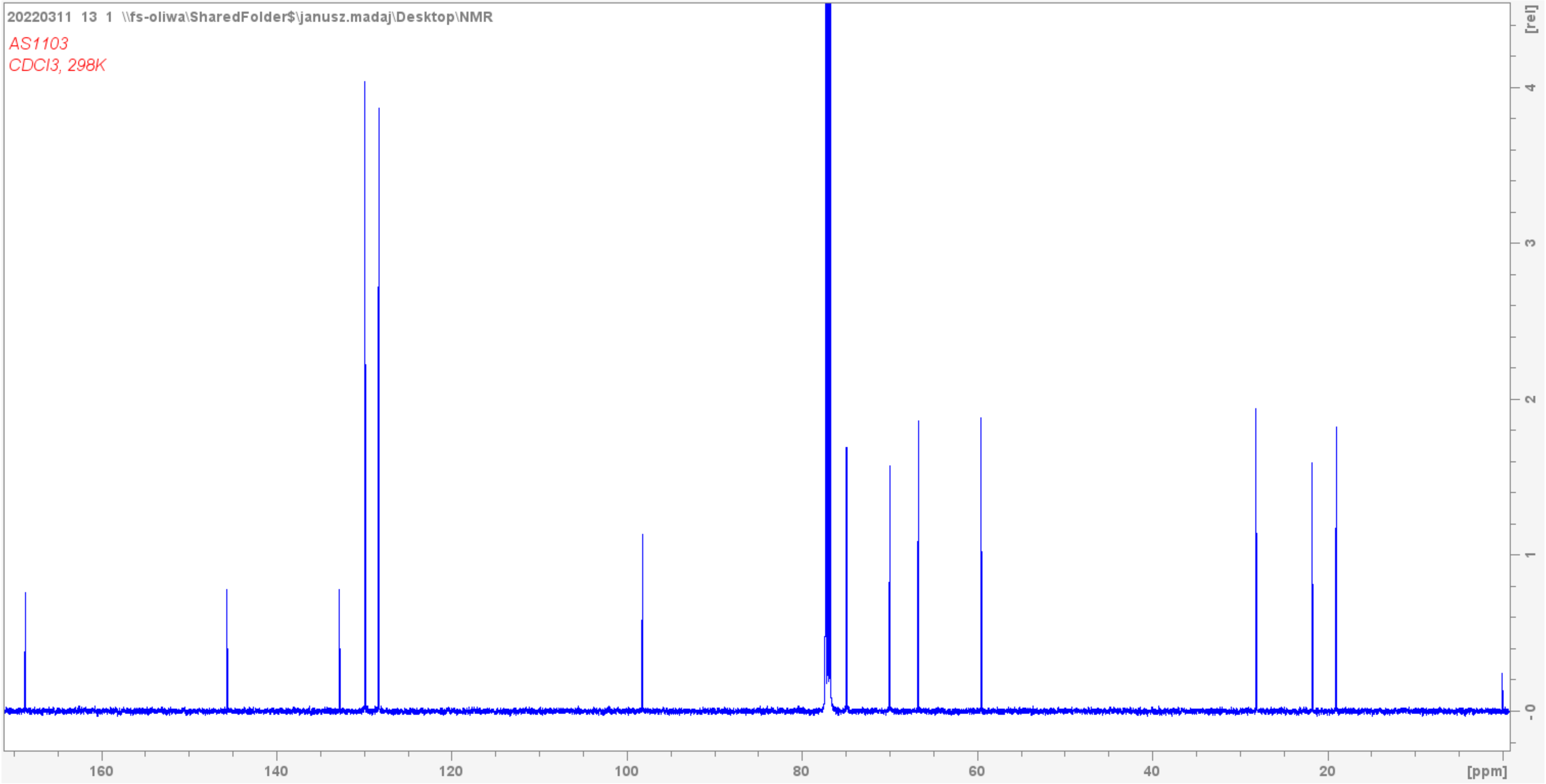

Fig. S7. ^{13}C NMR spectrum of 3,5-*O*-isopropylidene-2-*O*-tosyl- D-lyxono-1,4-lactone (**4**).

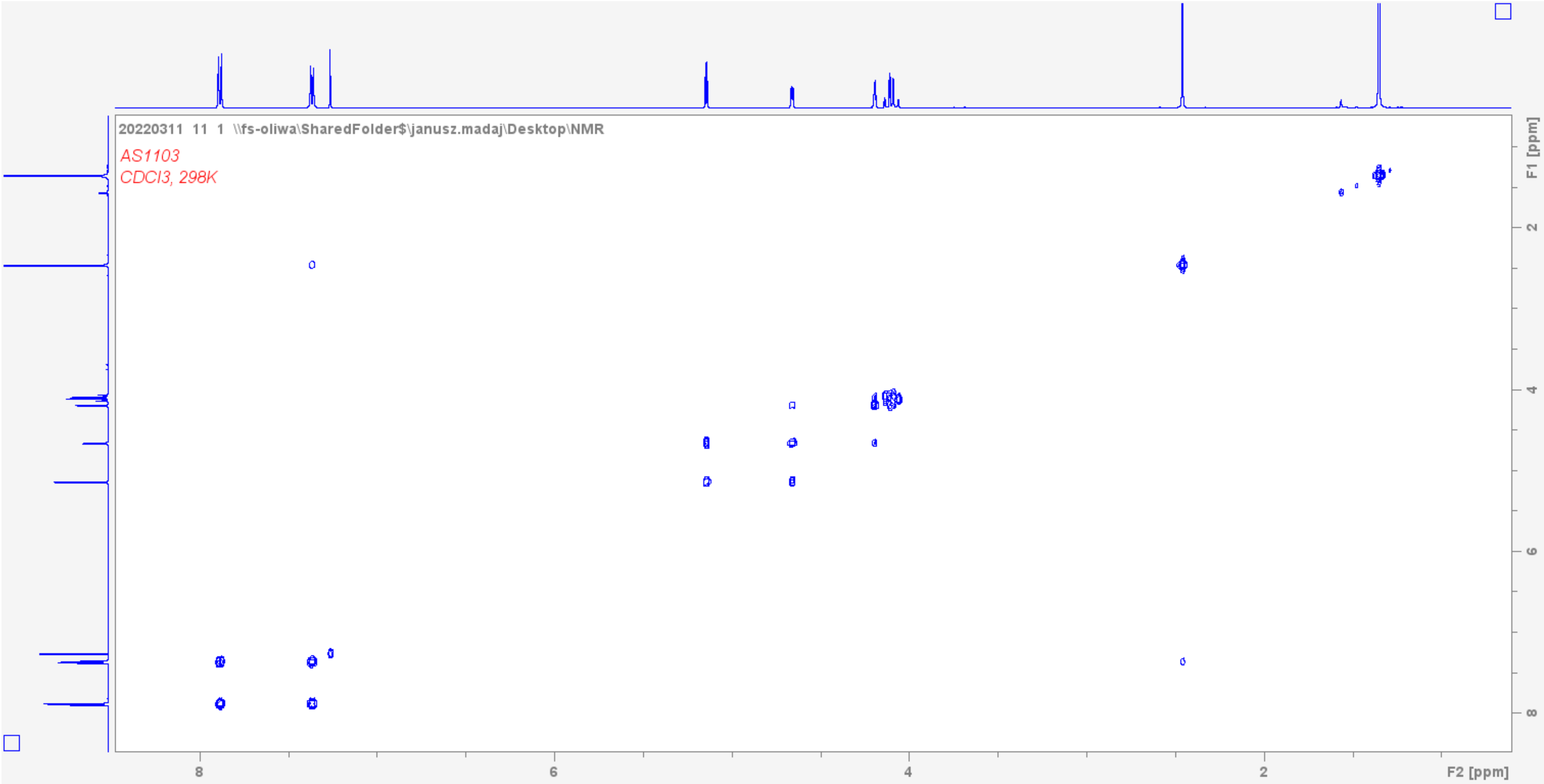

Fig. S8. COSY spectrum of 3,5-*O*-isopropylidene-2-*O*-tosyl- D-lyxono-1,4-lactone (**4**).

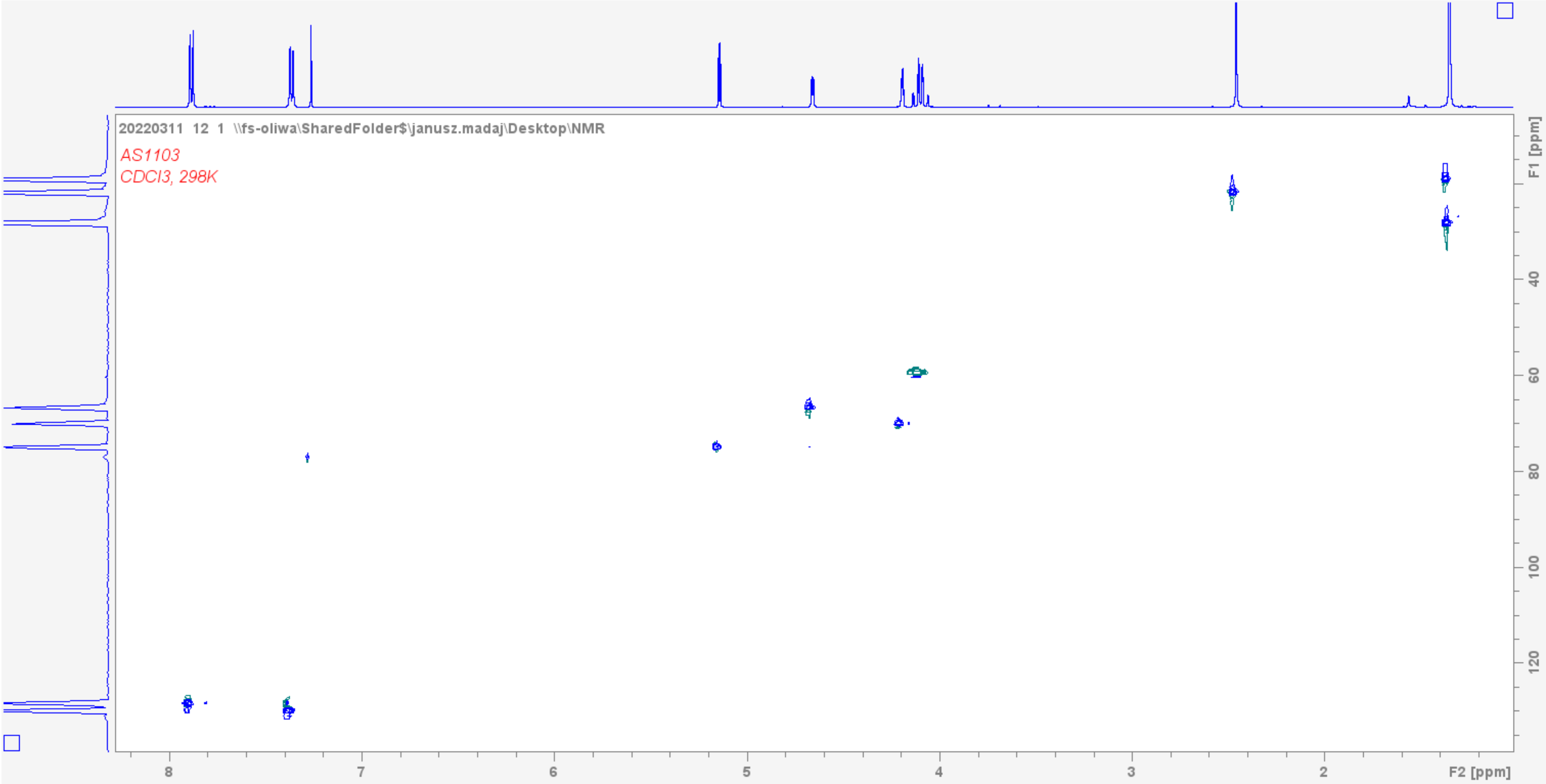

Fig. S9. HSQC spectrum of 3,5-*O*-isopropylidene-2-*O*-tosyl- D-lyxono-1,4-lactone (**4**).

Table S1. Fractional atomic coordinates and isotropic or equivalent isotropic displacement parameters (Å^2) for **2a**

	x	*y*	*z*	U_{iso}*/U_{eq}
O1	0.9042 (3)	0.62217 (12)	0.50392 (10)	0.0198 (3)
O2	1.1138 (3)	0.48060 (12)	0.39168 (11)	0.0218 (3)
O3	0.7950 (3)	0.29945 (11)	0.50979 (12)	0.0210 (3)
H3	0.656 (6)	0.255 (3)	0.510 (3)	0.032*
O4	0.8727 (3)	0.45056 (12)	0.70875 (11)	0.0210 (3)
H4	0.882 (6)	0.379 (3)	0.714 (2)	0.031*
O5	1.0499 (3)	0.69926 (12)	0.72978 (11)	0.0244 (3)
H5	1.077 (6)	0.622 (3)	0.760 (2)	0.030 (6)*
C1	0.9341 (3)	0.50755 (15)	0.45900 (13)	0.0164 (3)
C2	0.7119 (3)	0.42446 (14)	0.50849 (15)	0.0160 (3)
H2	0.543047	0.432909	0.459526	0.019*
C3	0.6631 (3)	0.48393 (15)	0.62893 (14)	0.0164 (3)
H3A	0.477116	0.464356	0.660481	0.020*
C4	0.6917 (4)	0.62110 (15)	0.59564 (14)	0.0174 (3)
H4A	0.515631	0.649967	0.560241	0.021*

C5	0.7734 (4)	0.71105 (17)	0.69182 (15)	0.0216 (4)
H5A	0.744070	0.797005	0.663187	0.026*
H5B	0.651620	0.698138	0.760275	0.026*

Table S2. Atomic displacement parameters (Å^2) for **2a**

	U^{11}	U^{22}	U^{33}	U^{12}	U^{13}	U^{23}
O1	0.0271 (6)	0.0148 (5)	0.0175 (5)	-0.0027 (4)	0.0008 (5)	0.0011 (4)
O2	0.0210 (6)	0.0244 (6)	0.0199 (5)	0.0011 (5)	0.0010 (4)	0.0004 (5)
O3	0.0174 (5)	0.0114 (5)	0.0343 (6)	0.0001 (4)	-0.0001 (5)	-0.0023 (5)
O4	0.0264 (6)	0.0161 (6)	0.0206 (6)	0.0017 (5)	-0.0069 (5)	0.0035 (4)
O5	0.0275 (6)	0.0200 (6)	0.0258 (6)	-0.0010 (5)	-0.0068 (5)	-0.0028 (5)
C1	0.0183 (7)	0.0148 (7)	0.0161 (6)	-0.0001 (6)	-0.0027 (6)	0.0013 (5)
C2	0.0151 (7)	0.0122 (7)	0.0208 (7)	0.0010 (5)	-0.0026 (6)	-0.0012 (5)
C3	0.0176 (7)	0.0138 (6)	0.0178 (7)	0.0002 (5)	-0.0013 (6)	0.0011 (5)
C4	0.0207 (7)	0.0138 (6)	0.0176 (7)	0.0015 (6)	-0.0018 (6)	0.0003 (6)
C5	0.0268 (8)	0.0167 (7)	0.0211 (7)	0.0009 (6)	-0.0019 (6)	-0.0040 (6)

Table S3. Geometric parameters (Å, º) for **2a**

O1—C1	1.3417 (19)	O5—C5	1.425 (2)
O1—C4	1.473 (2)	C1—C2	1.515 (2)
O2—C1	1.201 (2)	C2—C3	1.530 (2)
O3—C2	1.4038 (18)	C3—C4	1.529 (2)
O4—C3	1.416 (2)	C4—C5	1.514 (2)
C1—O1—C4	109.84 (12)	O4—C3—C2	110.81 (13)
O1—C1—C2	108.76 (13)	O4—C3—C4	109.69 (14)
O2—C1—O1	122.94 (15)	C4—C3—C2	99.60 (12)
O2—C1—C2	128.29 (15)	O1—C4—C3	104.34 (13)
O3—C2—C1	111.12 (13)	O1—C4—C5	108.71 (14)
O3—C2—C3	115.81 (14)	C5—C4—C3	117.47 (14)
C1—C2—C3	101.43 (12)	O5—C5—C4	114.37 (15)
O1—C1—C2—O3	-153.50 (13)	C1—O1—C4—C3	18.27 (17)
O1—C1—C2—C3	-29.83 (15)	C1—O1—C4—C5	144.37 (14)
O1—C4—C5—O5	-47.16 (19)	C1—C2—C3—O4	-77.24 (15)
O2—C1—C2—O3	25.8 (2)	C1—C2—C3—C4	38.21 (14)
O2—C1—C2—C3	149.47 (16)	C2—C3—C4—O1	-35.03 (15)
O3—C2—C3—O4	43.17 (18)	C2—C3—C4—C5	-155.43 (15)

O3—C2—C3—C4	158.63 (14)	C3—C4—C5—O5	70.9 (2)
O4—C3—C4—O1	81.28 (15)	C4—O1—C1—O2	-171.94 (15)
O4—C3—C4—C5	-39.1 (2)	C4—O1—C1—C2	7.40 (17)

Table S4. Fractional atomic coordinates and isotropic or equivalent isotropic displacement parameters (Å^2) for compound **4**.

	x	*y*	*z*	U_{iso}*/U_{eq}
S1	0.8081 (2)	0.60043 (9)	0.51560 (8)	0.0301 (4)
O1	0.4175 (7)	0.7850 (2)	0.7037 (2)	0.0310 (10)
O2	0.7705 (8)	0.8169 (3)	0.6660 (3)	0.0468 (13)
O3	0.5112 (7)	0.5961 (3)	0.7095 (2)	0.0295 (9)
O4	0.2723 (8)	0.6399 (3)	0.8072 (2)	0.0366 (10)
O5	0.7753 (7)	0.6532 (2)	0.5934 (2)	0.0285 (9)
O6	0.7034 (8)	0.6491 (3)	0.4553 (2)	0.0373 (10)
O7	1.0442 (7)	0.5851 (3)	0.5137 (3)	0.0392 (11)
C1	0.6042 (11)	0.7734 (4)	0.6611 (4)	0.0314 (13)
C2	0.5640 (10)	0.6954 (4)	0.6077 (3)	0.0270 (12)
H2	0.497166	0.716690	0.558623	0.032*
C3	0.3910 (10)	0.6422 (4)	0.6516 (3)	0.0275 (12)
H3	0.301023	0.601973	0.618164	0.033*
C4	0.2495 (10)	0.7170 (4)	0.6855 (3)	0.0283 (12)
H4	0.138877	0.739586	0.647155	0.034*
C5	0.1305 (11)	0.6908 (4)	0.7582 (3)	0.0340 (14)
H5A	0.081140	0.744760	0.785450	0.041*
H5B	-0.005678	0.656005	0.745412	0.041*
C6	0.3721 (11)	0.5663 (4)	0.7707 (3)	0.0328 (13)
C7	0.5334 (13)	0.5255 (4)	0.8275 (4)	0.0402 (15)
H7A	0.610965	0.475339	0.803758	0.060*
H7B	0.449124	0.504988	0.872163	0.060*
H7C	0.644735	0.569707	0.843289	0.060*
C8	0.1960 (12)	0.5004 (4)	0.7430 (4)	0.0389 (15)
H8A	0.094119	0.529535	0.706925	0.058*
H8B	0.109220	0.478485	0.786675	0.058*
H8C	0.271365	0.450790	0.717745	0.058*
C9	0.6602 (11)	0.5020 (4)	0.5312 (3)	0.0313 (13)
C10	0.4815 (11)	0.4797 (4)	0.4844 (4)	0.0352 (13)
H10	0.440789	0.516422	0.442690	0.042*

C11	0.3627 (12)	0.4025 (4)	0.4994 (4)	0.0412 (15)
H11	0.239468	0.386742	0.467553	0.049*
C12	0.4206 (13)	0.3477 (4)	0.5604 (4)	0.0389 (15)
C13	0.6050 (14)	0.3713 (4)	0.6048 (4)	0.0485 (19)
H13	0.648536	0.334223	0.646008	0.058*
C14	0.7265 (15)	0.4472 (4)	0.5905 (4)	0.0489 (19)
H14	0.853769	0.461845	0.620911	0.059*
C15	0.2898 (15)	0.2656 (5)	0.5771 (5)	0.0531 (19)
H15A	0.128399	0.279547	0.578546	0.080*
H15B	0.336688	0.241735	0.626544	0.080*
H15C	0.318416	0.221777	0.537140	0.080*

Table S5. Atomic displacement parameters (Å^2) for compound **4**.

	U^{11}	U^{22}	U^{33}	U^{12}	U^{13}	U^{23}
S1	0.0314 (8)	0.0346 (7)	0.0245 (7)	0.0019 (6)	0.0005 (6)	-0.0013 (6)
O1	0.032 (2)	0.028 (2)	0.033 (2)	0.0010 (17)	-0.0002 (19)	-0.0036 (17)
O2	0.032 (2)	0.034 (2)	0.074 (3)	-0.004 (2)	0.004 (3)	-0.014 (2)
O3	0.032 (2)	0.0304 (19)	0.026 (2)	0.0038 (17)	-0.0018 (17)	0.0031 (16)
O4	0.041 (2)	0.043 (2)	0.026 (2)	0.007 (2)	0.0014 (19)	0.0043 (17)
O5	0.030 (2)	0.0290 (19)	0.026 (2)	0.0036 (16)	-0.0015 (18)	-0.0042 (16)
O6	0.043 (2)	0.045 (2)	0.025 (2)	0.000 (2)	-0.002 (2)	0.0068 (18)
O7	0.032 (2)	0.049 (3)	0.036 (2)	0.0062 (19)	0.002 (2)	-0.009 (2)
C1	0.032 (3)	0.026 (3)	0.036 (3)	0.005 (2)	0.005 (3)	-0.001 (2)
C2	0.028 (3)	0.028 (3)	0.025 (3)	0.005 (2)	-0.002 (2)	0.001 (2)
C3	0.031 (3)	0.029 (3)	0.023 (3)	0.000 (2)	-0.003 (2)	0.000 (2)
C4	0.028 (3)	0.030 (3)	0.027 (3)	-0.001 (2)	-0.001 (2)	0.001 (2)
C5	0.034 (3)	0.040 (3)	0.028 (3)	0.009 (3)	0.003 (3)	0.001 (2)
C6	0.038 (3)	0.034 (3)	0.026 (3)	-0.001 (3)	0.004 (3)	0.005 (2)
C7	0.049 (4)	0.041 (3)	0.031 (3)	0.005 (3)	0.001 (3)	0.010 (3)
C8	0.041 (4)	0.034 (3)	0.042 (3)	-0.004 (3)	0.001 (3)	0.006 (3)
C9	0.037 (3)	0.034 (3)	0.023 (3)	0.004 (2)	-0.004 (2)	-0.003 (2)
C10	0.037 (3)	0.038 (3)	0.030 (3)	0.007 (3)	-0.004 (3)	0.003 (3)
C11	0.038 (3)	0.043 (3)	0.043 (4)	-0.003 (3)	-0.006 (3)	0.004 (3)
C12	0.049 (4)	0.031 (3)	0.037 (3)	0.005 (3)	0.005 (3)	-0.001 (2)
C13	0.079 (5)	0.031 (3)	0.036 (4)	-0.002 (4)	-0.015 (4)	0.003 (3)
C14	0.065 (5)	0.042 (4)	0.039 (4)	-0.002 (3)	-0.027 (4)	-0.001 (3)
C15	0.061 (5)	0.039 (3)	0.060 (4)	-0.006 (4)	0.003 (4)	0.006 (3)

Table S6. Geometric parameters (Å, º) for compound **4**.

S1—O5	1.593 (4)	C6—C8	1.523 (9)
S1—O6	1.430 (4)	C7—H7A	0.9800
S1—O7	1.419 (5)	C7—H7B	0.9800
S1—C9	1.751 (7)	C7—H7C	0.9800
O1—C1	1.347 (8)	C8—H8A	0.9800
O1—C4	1.468 (7)	C8—H8B	0.9800
O2—C1	1.189 (8)	C8—H8C	0.9800
O3—C3	1.424 (7)	C9—C10	1.382 (9)
O3—C6	1.426 (7)	C9—C14	1.387 (9)
O4—C5	1.428 (7)	C10—H10	0.9500
O4—C6	1.415 (7)	C10—C11	1.389 (9)
O5—C2	1.428 (7)	C11—H11	0.9500
C1—C2	1.526 (8)	C11—C12	1.396 (9)
C2—H2	1.0000	C12—C13	1.390 (10)
C2—C3	1.515 (8)	C12—C15	1.493 (10)
C3—H3	1.0000	C13—H13	0.9500
C3—C4	1.529 (8)	C13—C14	1.380 (11)
C4—H4	1.0000	C14—H14	0.9500
C4—C5	1.510 (8)	C15—H15A	0.9800
C5—H5A	0.9900	C15—H15B	0.9800
C5—H5B	0.9900	C15—H15C	0.9800
C6—C7	1.513 (9)		
O5—S1—C9	103.4 (3)	O4—C6—C7	106.7 (5)
O6—S1—O5	108.7 (2)	O4—C6—C8	111.9 (5)
O6—S1—C9	109.7 (3)	C7—C6—C8	112.1 (5)
O7—S1—O5	102.9 (3)	C6—C7—H7A	109.5
O7—S1—O6	119.7 (3)	C6—C7—H7B	109.5
O7—S1—C9	111.0 (3)	C6—C7—H7C	109.5
C1—O1—C4	110.2 (4)	H7A—C7—H7B	109.5
C3—O3—C6	113.7 (5)	H7A—C7—H7C	109.5
C6—O4—C5	113.5 (5)	H7B—C7—H7C	109.5
C2—O5—S1	118.8 (4)	C6—C8—H8A	109.5
O1—C1—C2	108.2 (5)	C6—C8—H8B	109.5
O2—C1—O1	124.7 (5)	C6—C8—H8C	109.5

O2—C1—C2	127.1 (6)	H8A—C8—H8B	109.5
O5—C2—C1	108.5 (5)	H8A—C8—H8C	109.5
O5—C2—H2	109.9	H8B—C8—H8C	109.5
O5—C2—C3	116.5 (4)	C10—C9—S1	119.9 (5)
C1—C2—H2	109.9	C10—C9—C14	121.1 (6)
C3—C2—C1	101.8 (5)	C14—C9—S1	118.9 (5)
C3—C2—H2	109.9	C9—C10—H10	120.6
O3—C3—C2	106.5 (5)	C9—C10—C11	118.8 (6)
O3—C3—H3	112.7	C11—C10—H10	120.6
O3—C3—C4	111.1 (4)	C10—C11—H11	119.3
C2—C3—H3	112.7	C10—C11—C12	121.3 (7)
C2—C3—C4	100.1 (4)	C12—C11—H11	119.3
C4—C3—H3	112.7	C11—C12—C15	121.1 (7)
O1—C4—C3	103.4 (5)	C13—C12—C11	118.1 (6)
O1—C4—H4	110.6	C13—C12—C15	120.8 (6)
O1—C4—C5	108.5 (5)	C12—C13—H13	119.2
C3—C4—H4	110.6	C14—C13—C12	121.5 (6)
C5—C4—C3	113.0 (5)	C14—C13—H13	119.2
C5—C4—H4	110.6	C9—C14—H14	120.4
O4—C5—C4	112.0 (5)	C13—C14—C9	119.1 (7)
O4—C5—H5A	109.2	C13—C14—H14	120.4
O4—C5—H5B	109.2	C12—C15—H15A	109.5
C4—C5—H5A	109.2	C12—C15—H15B	109.5
C4—C5—H5B	109.2	C12—C15—H15C	109.5
H5A—C5—H5B	107.9	H15A—C15—H15B	109.5
O3—C6—C7	105.0 (5)	H15A—C15—H15C	109.5
O3—C6—C8	111.4 (5)	H15B—C15—H15C	109.5
O4—C6—O3	109.5 (5)		
S1—O5—C2—C1	-153.3 (4)	C1—C2—C3—C4	37.8 (5)
S1—O5—C2—C3	92.7 (5)	C2—C3—C4—O1	-37.2 (5)
S1—C9—C10—C11	-178.1 (5)	C2—C3—C4—C5	-154.3 (5)
S1—C9—C14—C13	177.6 (6)	C3—O3—C6—O4	-61.9 (6)
O1—C1—C2—O5	-149.6 (5)	C3—O3—C6—C7	-176.1 (5)
O1—C1—C2—C3	-26.3 (6)	C3—O3—C6—C8	62.4 (6)
O1—C4—C5—O4	-72.1 (6)	C3—C4—C5—O4	41.9 (7)
O2—C1—C2—O5	29.6 (8)	C4—O1—C1—O2	-177.0 (6)
O2—C1—C2—C3	153.0 (7)	C4—O1—C1—C2	2.2 (6)

O3—C3—C4—O1	75.1 (5)	C5—O4—C6—O3	61.3 (6)
O3—C3—C4—C5	-42.0 (7)	C5—O4—C6—C7	174.4 (5)
O5—S1—C9—C10	120.0 (5)	C5—O4—C6—C8	-62.7 (6)
O5—S1—C9—C14	-60.4 (6)	C6—O3—C3—C2	160.6 (5)
O5—C2—C3—O3	39.7 (6)	C6—O3—C3—C4	52.5 (6)
O5—C2—C3—C4	155.5 (5)	C6—O4—C5—C4	-52.2 (7)
O6—S1—O5—C2	40.0 (4)	C9—S1—O5—C2	-76.6 (4)
O6—S1—C9—C10	4.2 (6)	C9—C10—C11—C12	-0.1 (10)
O6—S1—C9—C14	-176.3 (6)	C10—C9—C14—C13	-2.9 (11)
O7—S1—O5—C2	167.8 (4)	C10—C11—C12—C13	-1.6 (10)
O7—S1—C9—C10	-130.3 (5)	C10—C11—C12—C15	178.9 (7)
O7—S1—C9—C14	49.2 (6)	C11—C12—C13—C14	1.1 (11)
C1—O1—C4—C3	22.6 (6)	C12—C13—C14—C9	1.1 (12)
C1—O1—C4—C5	142.8 (5)	C14—C9—C10—C11	2.4 (10)
C1—C2—C3—O3	-78.0 (5)	C15—C12—C13—C14	-179.5 (7)
